# Detection of *Lotmaria passim*, *Crithidia mellificae* and Replicative Forms of Deformed Wing Virus and Kashmir Bee Virus in the Small Hive Beetle (*Aethina tumida*)

**DOI:** 10.3390/pathogens10030372

**Published:** 2021-03-19

**Authors:** Antonio Nanetti, James D. Ellis, Ilaria Cardaio, Giovanni Cilia

**Affiliations:** 1 CREA Research Centre for Agriculture and Environment, Via di Saliceto 80, 40128 Bologna, Italy; antonio.nanetti@crea.gov.it (A.N.); ilaria.cardaio@crea.gov.it (I.C.); 2 Entomology and Nematology Department, University of Florida, 1881 Natural Area Dr., P.O. Box 110620, Gainesville, FL 32607-0620, USA; jdellis@ufl.edu

**Keywords:** honey bee, small hive beetle, invasive pest, trypanosomatids, honey bee virus, deformed wing virus, Kashmir bee virus, replicative virus, strand-specific RT-PCR

## Abstract

Knowledge regarding the honey bee pathogens borne by invasive bee pests remains scarce. This investigation aimed to assess the presence in *Aethina tumida* (small hive beetle, SHB) adults of honey bee pathogens belonging to the following groups: (i) bacteria (*Paenibacillus larvae* and *Melissococcus plutonius*), (ii) trypanosomatids (*Lotmaria passim* and *Crithidia mellificae*), and (iii) viruses (black queen cell virus, Kashmir bee virus, deformed wing virus, slow paralysis virus, sacbrood virus, Israeli acute paralysis virus, acute bee paralysis virus, chronic bee paralysis virus). Specimens were collected from free-flying colonies in Gainesville (Florida, USA) in summer 2017. The results of the molecular analysis show the presence of *L. passim*, *C. mellificae*, and replicative forms of deformed wing virus (DWV) and Kashmir bee virus (KBV). Replicative forms of KBV have not previously been reported. These results support the hypothesis of pathogen spillover between managed honey bees and the SHB, and these dynamics require further investigation.

## 1. Introduction

*Aethina tumida* (Murray 1867), the small hive beetle (SHB), is a coleopteran species belonging to the Nitidulidae family [1]. Native to Sub-Saharan Africa [2], it is a destructive, invasive pest of *Apis mellifera* (western honey bee) colonies [3], and it causes significant damage to brood, pollen, and honey stores [4]. Presently, the SHB is recorded in all continents except Antarctica [3,5,6,7,8], having reached North America in 1996; Australia in 2000; and, more recently, countries in Europe, South America, and Asia [9,10,11,12]. The SHB is an ecological generalist [4] and creates persistent populations in colonies in areas in which it has been introduced [13].

Honey bees are exposed to pests and pathogens belonging to different groups (viruses, bacteria, fungi, protists, mites, insects, etc.), some of which are responsible for severe health impairment and colony collapse [14,15,16,17]. Adult SHBs invade colonies, where they feed, thrive, and reproduce. This allows contact between SHBs and other bee pests and pathogens [18,19,20,21,22,23].

*Lotmaria passim* and *Crithidia mellificae* are two trypanosomatid species capable of colonizing the digestive system of honey bees [24,25]. The transmission is deemed to occur by the oral–fecal route [26,27], and the presence of infected faeces within the hive may promote the circulation of the parasite among worker bees [26]. Both pathogens are deemed to impact colony health by altering bee behavior, physiology, immune response, and lifespan [28,29,30,31]. Nevertheless, the details of their pathogenic effects are still not fully understood. *Lotmaria passim* has been described only recently [25], and it is presently acknowledged as the most prevalent *A. mellifera* trypanosomatid [32]. Infections have been reported in Asian, European, and South and North American colonies [8], whereas *C. mellificae* infections have been rarely observed [8,33,34,35].

Deformed wing virus (DWV) is a positive-sense ssRNA virus belonging to the Picornaviridae family within the Iflavirus genus [36,37]. Spread globally [32,36,37,38], three genetic variants have been acknowledged and identified as types A, B, and C [39,40]. Type A is by far the most widespread [40], and it may generate asymptomatic or symptomatic infections, the latter including deformed or missing wings, shortened abdomens, and premature bee death [36]. Generally, this virus is transmitted through puncture wounds produced by the ectoparasite *Varroa destructor* as it feeds on immature honey bees [41]. However, the infection may be transmitted horizontally by bee-to-bee contact, especially in cases of severe infections [42,43,44,45,46], curbicular pollen, bee products, and floral contamination [47,48,49].

Kashmir bee virus (KBV) is a positive-sense ssRNA virus of the Dicistroviridae family within the Cripavirus genus [50,51], considered endemic in North America and Australia [52,53] but rarely reported in Europe [54,55,56,57,58]. It is genetically related to acute bee paralysis virus (ABPV) [59], and the two may co-infect the same colony or the same individual bee [59,60]. Low viral titers are generally detected in subclinical colonies; however, viral replication may be triggered by the presence of stressors, including *A. tumida* infestations [46,52,60], with a lethal outcome for different honey bee stages [59,61,62]. Ingestion of contaminated brood food [49,59,63] and *Varroa* feeding behavior [64,65,66] may elicit the transmission of KBV infections.

Herein, we aimed to assess the presence of the abovementioned pathogens (*L. passim, C. mellificae*, KBV, and DWV) in addition to pathogenic bacteria (*Paenibacillus larvae* and *Melissococcus plutonius*) and other bee viruses (ABPV), Israeli acute paralysis virus (IAPV), black queen cell virus (BQCV), sacbrood virus (SBV), chronic bee paralysis virus (CBPV), and slow paralysis virus (SPV, major and minor)) in SHB specimens collected in Florida, USA in 2017. This is an important first step in determining the role SHBs may plan in the movement of pathogens between honey bee colonies.

## 2. Results

The investigated samples, coming from the same honey bee colony, tested positive for *C. mellificae*, *L. passim,* KBV, and DWV (Table 1). No amplicons were detected for *P. larvae, M. plutonious*, ABPV, IAPV, BQCV, SBV, CBPV, SPV major, and SPV minor in SHB individuals and the pool of SHBs.

One of the SHB individuals was negative for all pathogens, whereas the other nine tested positive for one or two of them. The SHB pool was positive for both trypanosomatid species and the two virus types.

In the SHB individuals, no significant difference was found in the prevalence between *C. mellificae* and *L. passim* positives (bilateral Fisher’s exact test: *p* = 0.675). No co-infections with the two were detected.

The frequencies of DWV- and KBV-positive individuals did not significantly differ (bilateral Fisher’s exact test: *p* = 0.070). Viral coinfections were found only in one individual SHB, representing a significantly lower proportion of the positives (bilateral Fisher’s exact test: *p* = 0.010).

**Figure 1:**
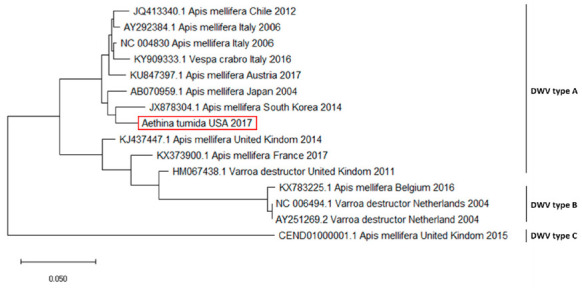
Molecular phylogenetic analysis for RNA-dependent RNA polymerase of deformed wing virus (DWV) using the maximum likelihood method. The evolutionary history was inferred using the maximum likelihood method based on the Tamura–Nei model. The branch lengths of the tree measured the number of substitutions per site. The analysis involved 28 nucleotide sequences. There were 255 positions in the final dataset. Accession number, host, state, and year of available GenBank DWV sequences are shown. DWV sequence accession numbers are reported and associated with year and site of origin and type. The DWV sequence obtained from the tested *Aethina tumida* samples is in a red box.

A strand-specific PCR demonstrated active viral replication of KBV and DWV in PCR-positive samples. Blast analysis on the sequences obtained from positive amplicons confirmed the specificity of the results, with high similarity (99%) to specific virus genome sequences deposited in GenBank. For each virus, the same sequence was recorded in all positive samples. Phylogenetic analysis and pairwise distance analysis indicated the highest homology to DWV type A (Figure 1).

**Figure 2:**
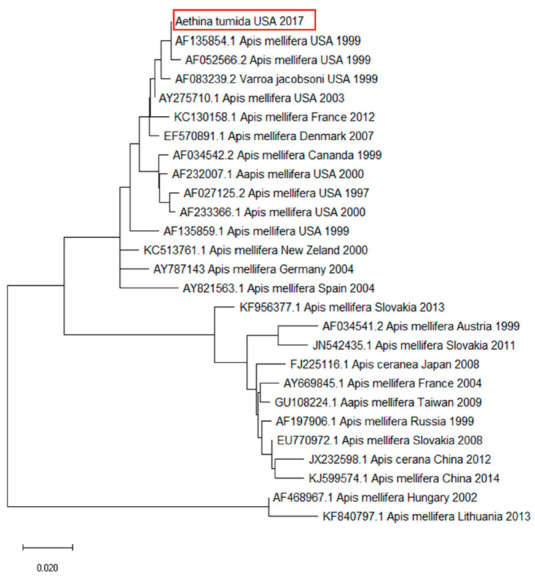
Molecular phylogenetic analysis for RNA-dependent RNA polymerase of Kashmir Bee Virus (KBV) using the maximum likelihood method. The evolutionary history was inferred using the maximum likelihood method based on the Tamura–Nei model. The branch lengths of the tree measured the number of substitutions per site. The analysis involved 35 nucleotide sequences. There were 297 positions in the final dataset. Accession number, host, state, and year of available GenBank KBV sequences are shown. KBV sequence accession numbers are reported and associated with the year and site of origin. The DWV sequence obtained from the tested *Aethina tumida* samples is in a red box.

A similar analysis was conducted for the KBV sequence. A close relationship with sequences found in *A. mellifera* and *V. destructor* from the USA was detected (Figure 2).

## 3. Discussion

To date, only a few instances of individual SHBs bearing bee pathogens have been reported. This is the case for samples from Mexico (positive for *L. passim*, *Apis mellifera* filamentous virus (AmFV), *C. bombi*, *Ascosphera apis*, and *Nosema ceranae* [22]), Florida (positive for *N. ceranae* [20]), and other areas of the USA (positive for DWV, SBV and *P. larvae* [19,20,22,24]).

The present study showed the presence of the honey bee pathogens *L. passim*, *C. mellificae*, DWV, and KBV in SHB adults collected from free-flying colonies in Florida. Furthermore, all SHB samples that were positive for DWV and/or KBV contained replicative viral forms. Although DWV replication in SHB adults is not a new finding [18,23], replication of KBV in SHBs is.

This is not the first time that DWV and KBV have been reported to infect non-Apis hosts. Replicative DWV was found in hornets (Vespa crabro) [67], Asian hornets (V. velutina) [68], and Argentine ants (Linepithema humile) [69]. Replicative KBV has been found in V. velutina [54], Vespula germanica, and Vespula vulgaris [70,71,72]. However, the current and previous [18,23] detections on coleopterans suggest that DWV and KBV can infect a wide range of potential hosts, thus envisaging a scenario where wild and managed insect species may act as virus reservoirs that fuel reciprocal spillover. Furthermore, the occurrence of replicative DWV and KBV in the same individuals indicates the possibility of viral co-infections in SHBs, as already reported in A. mellifera and other insect species [59,73,74].

The sequence analysis of DWV and KBV resulted in high identity rates to viral sequences identified in *A. mellifera*. The phylogenetic analysis highlighted that the DWV genome detected in the SHB samples belonged to DWV type A, the less virulent genetic variant of this virus [36]. The KBV genome found in the investigated samples bore a close relationship to other KBV outbreaks reported in the USA, thus excluding the involvement of viruses originating from other countries.

The prevalence of *L. passim*-positive individuals detected in this study (40% of all samples) mirrors that reported in a previous survey in which *C. bombi* was also reported in larval SHBs [22]. Additionally, we report for the first time SHB samples positive for *C. mellificae*. Although *C. mellificae* is generally considered less spread than *L. passim* in honey bees [33,34,35], the prevalence levels of the two trypanosomatids in our individual samples did not significantly differ.

None of the samples was positive for *P. larvae*, *M. plutonious*, ABPV, IAPV, BQCV, SBV, CBPV, SPV major, or SPV minor. This coincides with the results of previous investigations showing low *P. larvae* [19] and SBV [21] loads in SHB adults. This likely reflects the health of the colonies that were visited by the SHBs prior to sampling.

The finding results highlight the need to clarify pathogen transmission between honey bees and SHB adults better. In the case of DWV, horizontal transmission occurs chiefly by the oral route [18]. In this regard, SHBs are able to trick honey bee adults into feeding them [75,76], possibly acquiring DWV during the exchange of food via trophallaxis. However, the multifaceted host–parasite interaction [77] allows multiple pathways, including oral–oral and fecal–oral transmission. Adult SHBs also may acquire honey bee pathogens by feeding on bee products that are contaminated with multiple microorganism species [41,47,48,78], cannibalizing bee carcasses, or ingesting infected faeces [78,79,80].

The articulate interactions above and active flying behavior [8] may bring together adult SHBs of different origins that congregate in the same host colony, generating the detected diversity in the pathogen load. On the other hand, the horizontal transmission may occur bi-directionally, as both SHB adults and larvae might defecate inside the hive [77], potentially spreading infected feces that could transmit and perpetuate infective agents within the colony. Infections may also be transmitted vertically. Bee pathogens may be found in SHB larvae [22,23] as consequences of feeding, environmental contamination, and congenital transmission. Nevertheless, the role that SHBs play in the transmission of honey bee pathogens remains unclear.

## 4. Materials and Methods

### 4.1. Sample Collection

In summer 2017, one honey bee colony of mixed European origin was selected from an experimental apiary of the University of Florida (Gainesville, FL, USA) based on a conspicuous SHB infestation. No evident signs of other diseases could be detected. Forty SHB adults were randomly sampled alive from the colony combs and hive floor. Once in the laboratory, the collected specimens were randomly separated to compose one pool of thirty adults and ten individual beetle samples.

### 4.2. Extraction of Total Nucleic Acids

All the SHBs were washed with 95% ethanol to remove possible external microbial contaminants. The ethanol was then allowed to evaporate at room temperature.

A TissueLyser II (Qiagen, Hilden, Germany) was used for 3 min at 25 Hz to crush all SHB samples in separate 2 mL Eppendorf tubes filled to the mark with RNase-free water. The resulting suspensions were then split into two equal aliquots from which nucleic acids were extracted (one for DNA and one for RNA).

DNA and total RNA were extracted with DNeasy Blood & Tissue Kit (Qiagen) and RNeasy Mini Kit (Qiagen) as previously described [20,67]. All samples were eluted in 30 μL DNAase-RNase-free water.

DNA and RNA extracts were stored at −80 °C until analysis. High pure sterile DNA- and RNA-free water was used as a negative control in all analytical steps.

### 4.3. PCR Assays to Detect Bacteria and Protozoa DNA

The extracted DNA was analyzed by real-time PCR to detect bacteria and trypanosomatids. The primers that were used are reported in Table 2.

For each target gene, a total reaction volume of 15 μL was prepared as previously described [81] using 2x QuantiTect Probe PCR Master Mix (Qiagen), forward and reverse primers (2 μM), forward and reverse probes (500 nM), and 3 μL DNA extract. The real-time PCR assay was performed on a Rotorgene Corbett 6000 (Corbett Research, Sydney, Australia) following the protocols for either gene sequence [54,68]. DNA extracted previously from positive honey bees was used as the positive control for each investigated bacterial and protozoan species.

### 4.4. PCR Assays to Detect Virus RNA

All RNA extracts were retro-transcribed by M-MLV reverse transcriptase (Invitrogen, Carlsbad, CA, USA) using a blend of oligo-d (T) primers and random hexamers following the manufacturer’s instruction. Five microliters of the obtained cDNAs were used as a template for the PCR reactions, performed using HotStarTaqPlus Polymerase Mix (Qiagen). Primers to amplify the viral genomes of the honey bee viruses investigated herein are reported in Table 3. The real-time PCR assay was performed on a Rotorgene Corbett 6000. RNA extracted previously from positive honey bees was used as the positive control for each investigated virus.

### 4.5. Strand-Specific RT-PCR

To evaluate the replication of the detected viruses, strand-specific RT-PCRs were performed using specific primers, as previously described [47]. All cDNAs were amplified by PCR for the related viral target. The amplicons were detected on a 2% agarose gel, sequenced (BMR Genomics, Padua, Italy), and analyzed using BLAST [87]. Phylogenetic analysis was performed using the maximum likelihood method based on the Tamura–Nei model using MEGA software [88].

### 4.6. Statistical Analysis

The prevalence of the individuals that were positive for *C. mellificae* or *L. passim* and of those showing DWV or KBV infections were statistically compared with a bilateral Fisher’s exact test under the null hypothesis of equality. The same test was also used to compare the frequency of multiple vs. single viral infections. Due to the small number of samples, the test for independence χ^2^ was not used in this case.

## 5. Conclusions

This investigation suggests that the honey bee trypanosomatids *L. passim* and *C. mellificae* may colonize, and the viruses DWV and KBV successfully infect *A. tumida* adults. Additional studies are needed to determine whether these pathogens generate clinical evidence and signs of infection in SHBs. The horizontal and vertical transmission routes of these pathogens in/between SHBs should also be clarified, as well as the potential, if any, of these pathogens to limit SHB populations in the wild.

Finally, further research is needed to elucidate the epidemiological role that SHBs play in pathogen transmission to honey bees and other insects as a possible dead-end host or vector.

## Figures and Tables

**Table 1:** Summary of the *Aethina tumida* (SHB = small hive beetle) samples that tested positive for a given pathogen with the RT-PCR.

Target	Pool (*n* = 30)	SHB 1	SHB 2	SHB 3	SHB 4	SHB 5	SHB 6	SHB 7	SHB 8	SHB 9	SHB 10
***Crithidia. mellificae***	POS	-	-	POS	-	POS	-	-	POS	-	-
***Lotmaria passim***	POS	-	POS	-	POS	-	POS	-	-	POS	-
**KBV**	POS *	POS *	-	-	-	-	-	-	-	-	POS *
**DWV**	POS *	POS *	POS *	POS *	-	POS *	POS *	-	POS *	POS *	-

POS: positive; POS *: positive samples with replicative virus forms.

**Table 2:** List of primers used to detect bacteria and trypanosomatids in *Aethina tumida*.

Target	Primer Name	Sequence (5′-3′)	Reference
*Paenibacillus larvae*	AFB-F	CTTGTGTTTCTTTCGGGAGACGCCA	[82]
	AFB-R	TCTTAGAGTGCCCACCTCTGCG	
*Melissococcus plutonius*	MelissoF	CAGCTAGTCGGTTTGGTTCC	[83]
	MelissoR	TTGGCTGTAGATAGAATTGACAAT	
*Crithida mellificae*	Cmel_Cyt_b_F	TAAATTCACTACCTCAAATTCAATAACATAATCAT	[84]
	Cmel_Cyt_b_R	ATTTATTGTTGTAATCGGTTTTATTGGATATGT	
*Lotmaria passim*	Lp2F 459	AGGGATATTTAAACCCATCGAA	[33]
	Lp2R 459	ACCACAAGAGTACGGAATGC	

**Table 3:** List of primers used to detect viruses in Aethina *tumida*.

Target	Primer Name	Sequence (5′-3′)	Reference
KBV	KBV 83F	ACCAGGAAGTATTCCCATGGTAAG	[85]
	KBV 161R	TGGAGCTATGGTTCCGTTCAG	
DWV	DWV Fw 8450	TGGCATGCCTTGTTCACCGT	[47]
	DWV Rev 8953	CGTGCAGCTCGATAGGATGCCA	
ABPV	APV 95F	TCCTATATCGACGACGAAAGACAA	[85]
	APV 159R	GCGCTTTAATTCCATCCAATTGA	
IAPV	IAPV B4S0427_R130M	RCRTCAGTCGTCTTCCAGGT	[86]
	IAPV B4S0427_L17M	CGAACTTGGTGACTTGARGG	
BQCV	BQCV 9195F	GGTGCGGGAGATGATATGGA	[85]
	BQCV 8265R	GCCGTCTGAGATGCATGAATAC	
SBV	SBV 311F 79	AAGTTGGAGGCGCGyAATTG	[85]
	SBV 380R	CAAATGTCTTCTTACdAGAGGyAAGGATTG	
CBPV	CPV 304F 79	TCTGGCTCTGTCTTCGCAAA	[85]
	CPV 371R	GATACCGTCGTCACCCTCATG	
SPV major	SPV 8383F 81	TGATTGGACTCGGCTTGCTA	[59]
	SPV 8456R	CAAAATTTGCATAATCCCCAGTT	
SPV minor	SPV Minor F1	ATAGCGCTTTAGTTCAATTGCCAT	[38]
	SPV Minor R1	CTGGAATATGACCATCACGCAT	

KBV: Kashmir bee virus; DWV: Deformed wing virus; ABPV: Acute bee paraylis virus; IAPV: Israeli acute bee paryalis virus; BQCV: Black queen cell virus; SBV: Sac brood virus; CBPV: Chronic bee parylis virus; SPV: slow paralysis virus.
